# Tracheal reconstruction with nail grafts: A novel approach

**DOI:** 10.1016/j.xjtc.2021.10.032

**Published:** 2021-10-19

**Authors:** Hui-Fu Huang, Juey-Jen Hwang, Pei-Ming Huang

**Affiliations:** a Division of Plastic Surgery, Department of Surgery, National Taiwan University Hospital and National Taiwan University College of Medicine, Taipei, Taiwan; b Cardiovascular Center, National Taiwan University Hospital Yunlin Branch, Yunlin County, Taiwan; c Division of Cardiology, Department of Internal Medicine, National Taiwan University College of Medicine and Hospital, Taipei, Taiwan; d Department of Medicine and Surgery, Thoracic Medicine Center, National Taiwan University Hospital Yunlin Branch, Yunlin County, Taiwan; e Division of Thoracic Surgery, Department of Surgery, National Taiwan University Hospital and National Taiwan University College of Medicine, Taipei, Taiwan

**Keywords:** graft, nail, reconstruction, trachea, CT, computed tomography

## Abstract

**Background:**

The replacement of tracheal defects has been a challenge for investigators worldwide. We aimed to develop autologous nail grafts for the reconstruction of anterior tracheal defects.

**Methods:**

Toenail grafts were implanted to improve the structural integrity of the trachea in patients with tracheal diseases. We clinically applied these grafts for the partial replacement of the cervical tracheal cartilage. Data on graft construction details, clinical outcomes, bronchoscopy, and 3-dimensional computed tomography examinations were collected.

**Results:**

The nail grafts were implanted in 4 patients. The trachea was successfully reconstructed in all cases. Bronchoscopy was performed 3 times to document healing: immediately, 1 month, and 3 months after surgery. All grafts were well vascularized and incorporated into the tracheal wall and were covered with the respiratory mucosa. Three of the patients survived during the study period, but 1 patient died of progressive lung cancer.

**Conclusions:**

Toenail grafts potentially may be used as an alternative strategy for the closure of small defects during tracheal reconstruction.

## Graphical abstract

**Figure:**
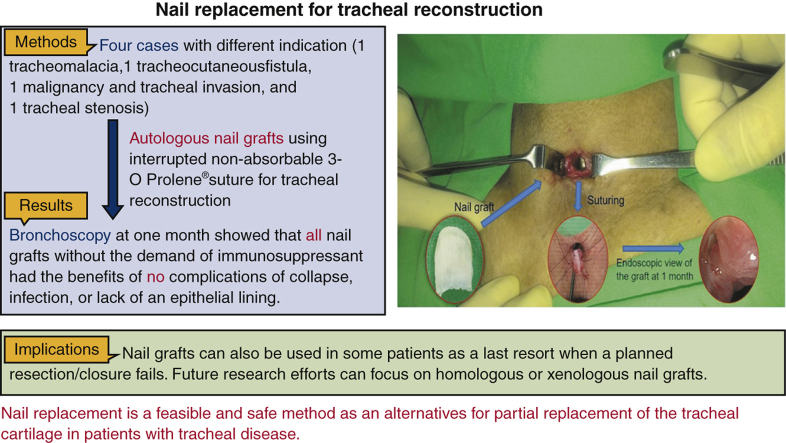

**Figure:**
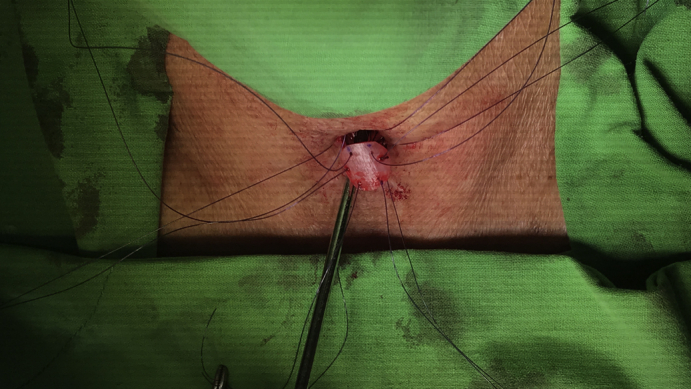
A nail graft was applied for the partial replacement of a cervical tracheal defect.

**Central Message**

Implanting nail grafts can be an alternative to partial replacement of tracheal cartilage in patients with tracheal disease.

**Perspective**

The replacement of major defects of the trachea remains a challenge despite numerous attempts to use different grafts. Nail grafts are applicable for tracheal reconstruction because they provide rigid support and are not associated with long-term complications such as collapse, infection, or the lack of an epithelial lining.

See Commentary on page 561.

The adult trachea can be resected with primary anastomosis in most cases; however, longer resections are at greater risk for anastomotic complications. Replacement of the tracheal defect remains a challenge despite numerous attempts to use different grafts, which have considerably evolved over time. Tracheal reconstruction requires the creation of a rigid fibrocartilaginous support. Unfortunately, different alternatives comprising either biological or artificial grafts have been used for tracheal anastomosis with little clinical success.^1^, ^2^, ^3^ Three main techniques of tracheal reconstruction have been described in the literature, including synthetic matrix graft, free-tissue transfer, and allotransplantation. The challenges in tissue engineering of thin hollow organs include incorporating the neo-tissues into the existing structures and preventing the formation of a stricture.^4^, ^5^, ^6^ The survival of these nonvascularized tissue-engineered grafts over a long period is also uncertain. Several studies have attempted to create cartilage using mesh sheets for tracheal reconstruction. Unfortunately, these grafts eventually degenerate, resulting in poor outcomes due to graft collapse, stenosis, further infection, or lack of an epithelial lining leading to rejection.^2^^,^^7^^,^^8^ A number of other techniques proven to be reproducible and effective (eg, use of aortic homograft or vascularized autografts) have been recently reported.^9^^,^^10^

Toenail grafts are applicable in tracheal reconstruction due to their natural physiologic and anatomic structures, which retain the flexibility and lumen patency of the respiratory tract. In this study, we report the outcomes of implantation using an autologous toenail graft as a substitute for tracheal reconstruction.

## Materials and Methods

We retrospectively reviewed the medical records and radiologic investigations of 4 patients aged 55 to 75 years who had received preoperatively planned autologous toenail grafts for tracheal reconstruction between June 2017 and October 2019; 1 had tracheomalacia, 1 had tracheocutaneous fistula, 1 had malignancy and tracheal invasion, and 1 had tracheal stenosis. Pretreatment evaluation included medical history, physical examination, complete blood count, biochemistry survey, neck and chest computed tomography (CT), and bronchoscopy. This study was approved by the Institutional Review Board of the National Taiwan University Hospital (NTUHREC 202005077RINA). The patients provided written informed consent for each follow-up investigation.

### Preparation of the Big Toe Nail Graft

Surgical nail avulsion is a simple and frequently performed procedure for diagnostic or therapeutic purposes. After the administration of general anesthesia and the application of beta-iodine to the big toe, a Freer elevator was used to easily develop an avascular plane below the nail plate without bleeding. The instrument was introduced under the distal free edge of the nail plate, so that the nail plate could be separated from the underlying nail bed hyponychium. The nail plate was then separated from the underlying nail bed and directed proximally toward the nail matrix. A lateral sweeping movement was performed until the instrument reached the distal edge of the nail plate. The nail graft was then removed using a platypus nail puller. The nail graft was measured on a line across its longest horizontal and vertical axes. Finally, antiseptic normal saline irrigation was used to reduce the bacterial contamination of the vascular nail bed. The nail donor site was then covered with a Vaseline-immersed dressing. After extraction of the nail graft, the external side of the big toenail lining was molded for further tracheal reconstruction.

### Tracheal Reconstruction

**Figure 1:**
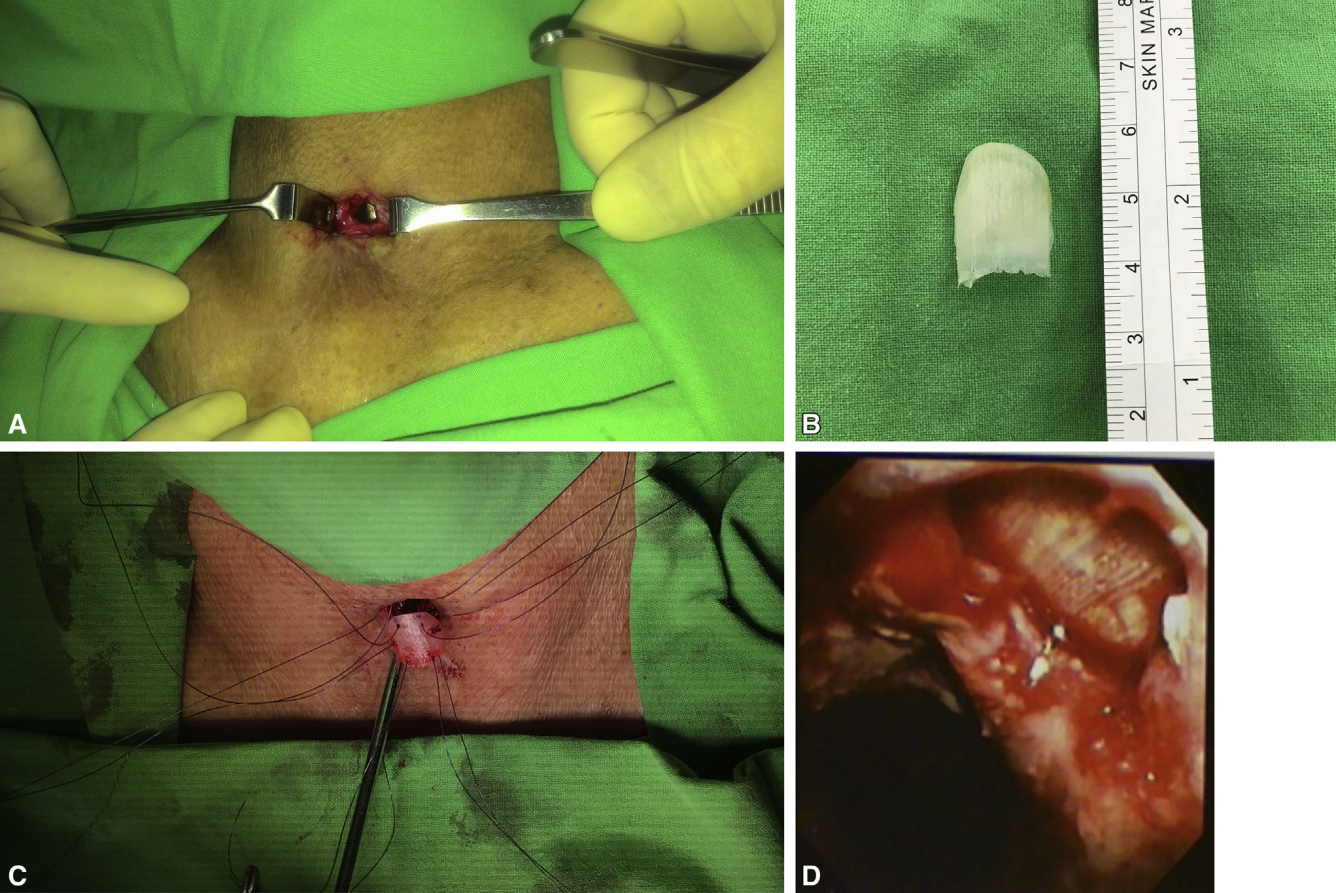
Partial tracheal defect reconstruction with nail graft in the second patient. A, The anterior wall of the cervical tracheal defect exposed through a vertical neck incision. B, Nail avulsion accomplished using an elevator and nail puller. A 2.2 × 1.6-cm big toenail was harvested, and the external surface of the big toenail lining was shaped for the tracheal reconstruction. C, Next, the nail graft was reoriented and sutured to the margins of the tracheal defect using simple interrupted 3-0 Prolene sutures. D, Intraoperative bronchoscopy showed that the tracheal defect had closed over the nail graft to augment its integrity.

During the second stage of the operation, a longitudinal anterior tracheal defect was covered with a toenail for patients 1, 2, and 4 (Video 1 shows patient 2) and a 1-cm lateral tracheal defect after the resection of metastatic lymph nodes in patient 3. For the anterior defects, the cervical trachea was exposed through a vertical neck incision to visualize the surface of the tracheal cartilage (Figure 1, *A*). The anterior tracheal wall was incised under bronchoscopic guidance to identify the stenotic segment of the trachea. The nail graft was subsequently transposed to the neck. The nail was naturally curved, which permitted the easy reconstruction of the anterior circular defect (Figure 1, *B*). The graft was then reoriented to allow its concave surface to accommodate the curved surface of the ventral side of the nail plate. Afterward, the nail flap was sutured to the margins of the tracheal defect using simple interrupted nonabsorbable 3-0 Prolene (Ethicon; Surgipro: USSDG, Norwalk, Conn) sutures (Figure 1, *C*). To begin the closure, the strap muscles were repaired at the anastomotic site over the graft in the midline. Irrigation with gentamycin was then performed before the insertion of a Jackson–Pratt drain tube. Finally, the skin was sutured snugly using a 3-0 nylon buttress at 1-cm slack intervals to allow air spillage and prevent subcutaneous emphysema. All patients were successfully extubated on the operating room table after the intraoperative bronchoscopic evaluation (Figure 1, *D*).

**Video 1:**
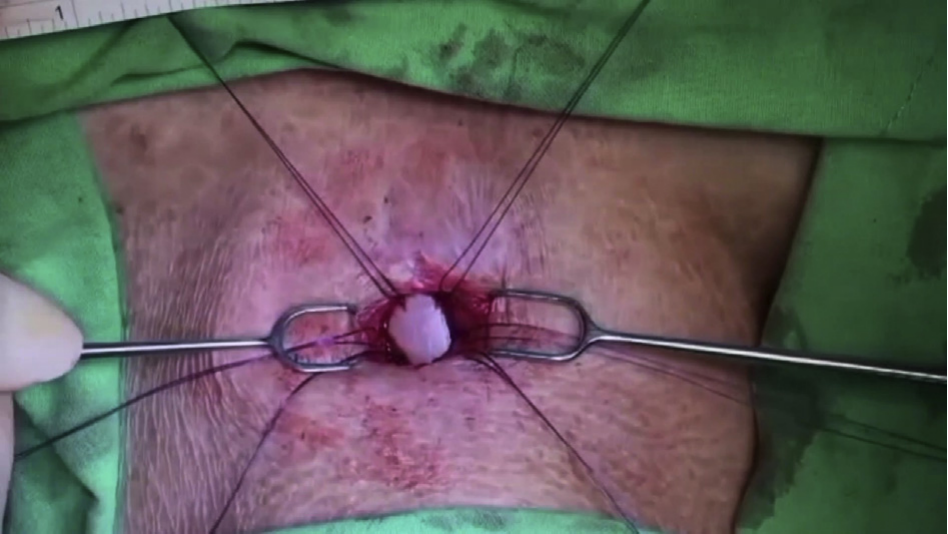
Operative procedure of using a nail graft for tracheal reconstruction. After the extraction of the nail graft, it was transposed to the neck. The nail graft was marked at 6 points and then reoriented to allow its concave surface to accommodate the curved surface of the trachea. The nail graft was then sutured to the margins of the tracheal defect using simple interrupted 3-0 Prolene sutures. Video available at: https://www.jtcvs.org/article/S2666-2507(21)00723-9/fulltext.

### Postoperative Monitoring and Assessment

**Figure 2:**
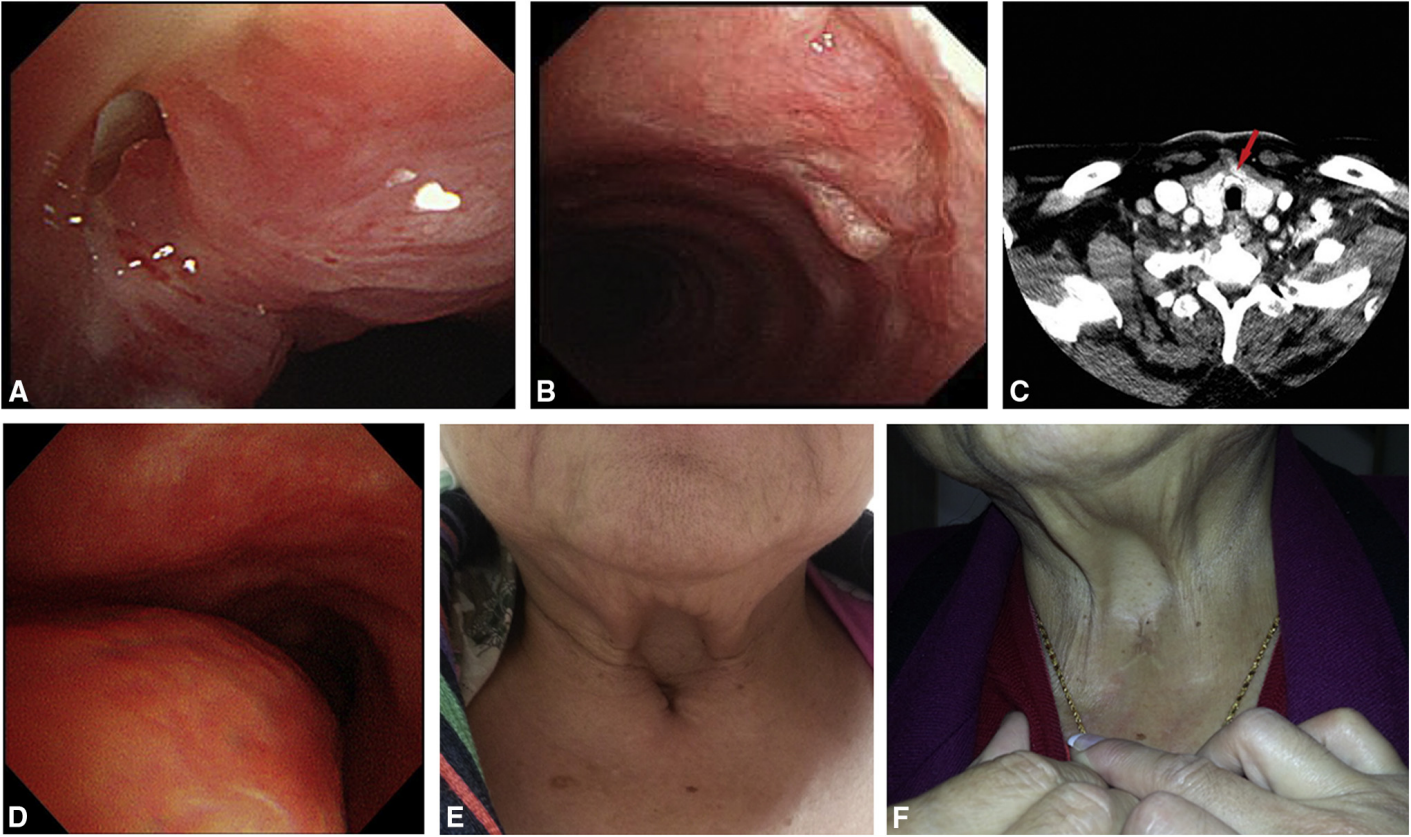
Postoperative assessment. A and B, Endoscopic zoom in and zoom out perspective views of the graft at 1 month postoperatively in the second patient, showing the nail graft well integrated with the native cartilage rings and covered with respiratory epithelium. C, Neck CT of the fourth patient shows calcification of the nail graft (*red arrow*) and no impending tracheal obstruction at 3 months postoperatively. D, Preoperative bronchoscopy of the first patient reveals bulging of the membranous portion of the trachea. E, The first patient initially had tracheomalacia and retraction of the skin over the frontal part of the neck. F, At the most recent follow-up, the cosmetic appearance of the neck and airway symptoms have improved after the reconstruction.

Postoperatively, the patients were monitored both clinically and endoscopically. They were inspected daily for wound dehiscence or infection, and endoscopy was performed at 1- and 3-month intervals after surgery (Figure 2, *A* and *B*). Flexible bronchoscopy was performed to assess the graft and obtain biopsy samples to assess the regeneration of the bronchial mucosa. Subsequently, the patients were followed up for assessment of the graft properties using CT (Figure 2, *C*) and virtual bronchoscopic images.

## Results

The mean hospital stay of the patients was 11 ± 7.8 days, and the mean follow-up period was 16.7 months (Table 1). Bronchoscopy at 1 month postoperatively revealed a patent airway compared with the normal area of the trachea, with no paradoxical movements of the tracheal wall. The nail graft was well integrated with the native cartilage rings (Figure 2, *A* and *B*). None of the patients developed wound dehiscence, infection, or erosion; no cicatricial stenosis developed in the nail graft area approximating the tracheal anastomosis. The nail graft also appeared to be lined with respiratory epithelium, and normal mucus clearance was observed (Figure 2, *A* and *B*). Activities of daily living and cough reflex were normal in all patients during the postoperative follow-up at the outpatient clinic. No surgical mortalities were observed. At 3 months postoperatively, neck CT revealed calcification of the reconstructed airway (Figure 2, *C*). The tracheal defects were repaired with the nail graft, which was adequate for tracheal reconstruction, exhibiting good intermediate-term patency.

**Table 1:** Patient and autologous nail tracheal graft characteristics

	Patient 1	Patient 2	Patient 3	Patient 4
Gender	Female	Male	Female	Male
Age, y	75	68	55	62
Underlying disease	Asthma, CAD, DM	End-stage renal disease, CAD, DM	Stage IV lung adenocarcinoma	Heart failure, CKD, CAD
Etiology of tracheal disease	Skin retraction and tracheomalacia due to tracheostomy	Tracheocutaneous fistula 1 cm in diameter for 1 y due to tracheostomy	Resection of lymphadenopathy with tracheal compression	Tracheal stenosis due to previous tracheostomy
Nail graft, cm	2.2 × 1.6	2.5 × 2.0	2.5 × 1.5	2.6 × 2.1
Postoperative bronchoscopic intervention, 1 mo	No obstruction, no collapse, no breakdown	No granulation, no collapse, no breakdown	No obstruction, no collapse, no breakdown	Granulation at the suture line, no collapse, no breakdown
Adverse events	None	None	None	None
Hospital stay	17 d	2 d	7 d	18 d
Follow-up periods, d	1026	733	61	183
Outcomes	Occasional asthma attack	No airway symptom	Died with cancer progress 2 mo after surgery	No airway symptom

*CAD*, Coronary artery disease; *DM*, diabetes mellitus; *CKD*, chronic kidney disease.

## Patient List

### Patient 1

The patient had tracheomalacia and skin retraction over the frontal part of the neck due to a previous tracheostomy performed for an asthmatic episode (with respiratory failure) approximately 4 years before the tracheal repair (Figure 2, *D* and *E*). During the most recent postoperative follow-up, the cosmetic appearance of the neck had improved, and the patient still had occasional asthma with minimal airway symptoms (Figure 2, *F*).

### Patient 2

**Figure 3:**
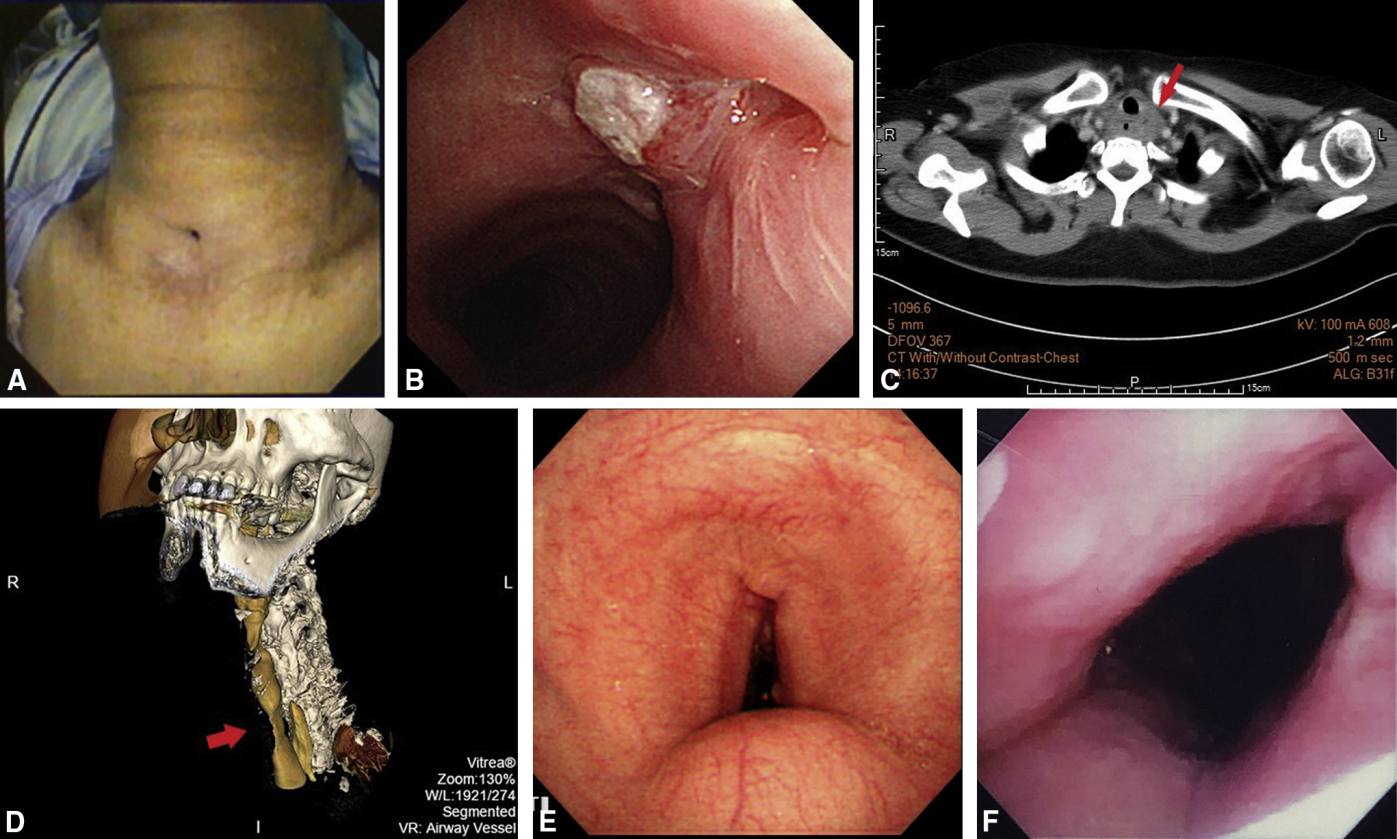
Several indications for the use of a nail graft for tracheal reconstruction. A, The patient had a persistent tracheocutaneous fistula after tracheostomy decannulation. B, Bronchoscopy reveals a persistent tracheocutaneous fistula. C, The patient had recurrent neck lymph node metastasis leading to tracheal compression (*red arrow*) after chemotherapy for lung adenocarcinoma. D, Preoperative 3-dimensional reconstruction CT shows tracheal stenosis (*red arrow*). E, Preoperative bronchoscopy of the fourth patient shows narrowing of the tracheal lumen with associated loss of the semicircular shape and granulation from previous tracheostoma defects. F, At 1 month postoperatively, bronchoscopy of the fourth patient shows improvement of postintubation stenosis.

The patient had poor wound closure of the tracheostoma after the removal of a tracheostomy tube (Figure 3, *A* and *B*). Sputum discharge from the tracheocutaneous fistula interrupted his speech. However, the patient's routine activities improved after the tracheal reconstruction with the nail graft.

### Patient 3

The patient originally underwent resection of a solitary recurrent neck lymph node metastasis, after chemotherapy for lung adenocarcinoma (Figure 3, *C*), for further analysis of genetic mutations. However, a 1-cm “hole punch” of the partial tracheal wall remained after the resection, which was covered with a big toenail graft to repair the tracheal defect.

### Patient 4

The patient had tracheal stenosis (Figure 3, *D* and *E*) due to a previous emergency tracheostomy, so a harvested big toenail was sutured to increase the total tracheal diameter without partial or segmental resection. Postoperative neck CT and bronchoscopy revealed the calcification of the reconstructed nail graft without airway stenosis (Figure 2, *C* and *F*).

## Discussion

Numerous studies have reported the use of grafts to repair tracheal defects.^11^^,^^12^ Three main concerns contribute to the unified problems of using tracheal grafts. The first concern is regarding stability, which affects airway patency. The rib or ear cartilage is used to prevent stenosis and maintain tracheal stability.^12^ However, these cartilage grafts may have resorption of the prosthetic material.^12^ The high failure rates of cartilage grafts are caused by ill-fitting mechanical properties and the closely tailored tracheal anatomy. The second concern is associated with cell adhesion, wherein the tracheal graft encounters slow epithelium formation on the inner lumen surface.^4^^,^^13^^,^^14^ To address this issue, photocrosslinked natural hydrogel was recently used to enhance the cell retention efficiency and improve the tracheal cartilage regeneration in a nude mouse study.^15^ The third concern is related to vascularization, which affects the ability of the graft to survive within the airway lumen and increases the risk of harboring microbes. Moreover, further research is necessary to reduce scarring at the anastomotic sites, particularly for graft prostheses that degrade and regenerate fibrotic properties.^16^

A nail graft is an available option that offers many advantages over other types of grafts. The new nail will regrow at the donor site in approximately 2 months. More important, the nail graft prosthesis achieves uniform thickness that approximates that of the natural trachea. Additionally, the nail graft is naturally curved and can be used to cap a tracheal defect for up to half of its circumference without any obstruction. In our nail graft design, it counteracted any contraction forces and prevented airway collapse induced by the negative inspirational forces. Although other patch flaps are available for tracheal defects, airway stenosis often develops due to patch contraction. The autographic biocompatibility and suitability of the nail graft for sustaining tracheal epithelial cell proliferation and differentiation were better than those seen with other grafts.^17^ The nail graft supports re-epithelialization, facilitated by focal repair mechanisms via cell seeding. Moreover, segmental tissue-engineered tracheal graft reconstructions more commonly lead to stenosis or delayed epithelialization when compared with nail graft patch tracheoplasty.^8^^,^^17^ The long-term stability of the nail graft was adequate, no nail graft necrosis due to lack of revascularization was observed, and the need to wrap the graft with a pedicle flap did not arise.^7^^,^^18^ Overall, nail grafts resist collapse and compression, which often limit the function of other tracheal grafts, and do not require any cells from the recipient. Moreover, nail grafts do not share the concern that autologous stem cells might give rise to tumors.

In this preliminary study, there were different indications reported for the use of a toenail graft, including persistent tracheocutaneous fistula, tracheomalacia, tumor invasion, and tracheal stenosis. Therefore, some common conclusions were difficult to generalize. Such defects may be treated with segmental resection, dilation, or airway stenting; however, these techniques have high rates of postoperative morbidity.^19^ Persistent tracheocutaneous fistula after decannulation of a tracheostomy is a known complication of long-term tracheostomy, which leads to several morbidities, including difficulty in vocalization, sputum secretion, airway infections, and poor cosmetic appearance. The simplest technique to avoid its formation is to either mobilize the straps muscles and use them to seal the small defect, or to create an autologous flap wherein the peri-stoma skin epithelium is turned in and reinforced by the strap muscles. Although other methods could have been used in our patients, nail grafts appear to be the better alternative choice. We report a new method for closing persistent tracheocutaneous fistulas with nail grafts to prevent potential airway obstruction proximal to the turn in the peristomal skin epithelium. Essentially, we also used a nail graft to restore lung function in a patient with severe tracheomalacia.

### Study Limitations

**Figure 4:**
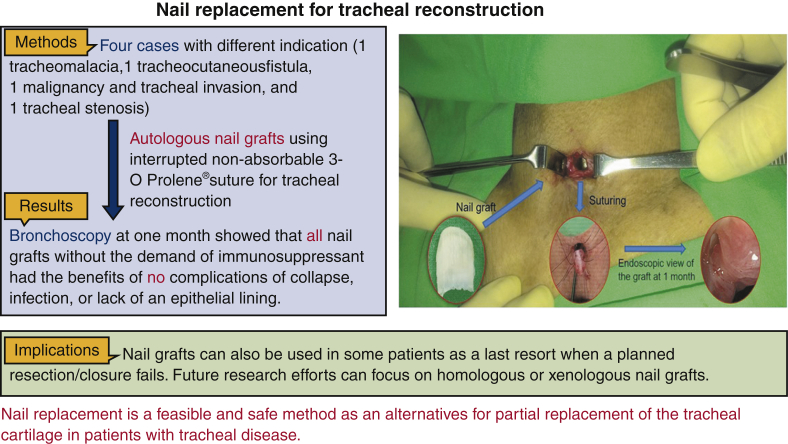
This study developed autologous nail grafts for the reconstruction of anterior tracheal defects. Four patients received autologous nail grafts for their tracheal reconstruction. The nail graft was transposed into the neck and then sutured to the margins of the tracheal defect using simple interrupted nonabsorbable sutures. Bronchoscopy showed patent grafts well integrated with the native cartilage rings at 1 month postoperatively.

In the future, nail grafts may be used as a last resort for the tracheal repair in some patients with failed resections or closures. Future research efforts should focus on homologous or xenologous nail grafts. This could presumably be performed even if a patient is a bilateral amputee. However, despite these promising preliminary results, concerns regarding the long-term ability and viability of a nail graft in the airway as well as the limitation of its length remain. Our study is also limited by the relatively small number of patients with complete follow-up evaluations, the single-institutional setting, and the retrospective study design. We look forward to follow-up investigations with longer observation periods to better evaluate the results of our findings. We concede the difficulties encountered in the circumferential replacement of the tracheal airways, and further improvements in our technique are necessary before our design can be used as a routine technique. Finally, patient-specific responses following reconstruction must be optimized individually. There were different indications for the use of toenail grafts; therefore, some common conclusions remain difficult to generalize. Further follow-ups, standardization of indications, and a larger sample size are necessary to validate this method of reconstructive surgery (Figure 4).

## Conclusions

These initial clinical results provide evidence that the use of a nail graft for tracheal reconstruction shows some potential as an alternative means for closing small tracheal defects; however, additional investigations are required to further validate its long-term ability and viability.

### Conflict of Interest Statement

The authors reported no conflicts of interest.

The *Journal* policy requires editors and reviewers to disclose conflicts of interest and to decline handling or reviewing manuscripts for which they may have a conflict of interest. The editors and reviewers of this article have no conflicts of interest.

## Supplementary Data

Video 1 Operative procedure of using a nail graft for tracheal reconstruction. After the extraction of the nail graft, it was transposed to the neck. The nail graft was marked at 6 points and then reoriented to allow its concave surface to accommodate the curved surface of the trachea. The nail graft was then sutured to the margins of the tracheal defect using simple interrupted 3-0 Prolene sutures. Video available at: https://www.jtcvs.org/article/S2666-2507(21)00723-9/fulltext.
